# Image quality and detectability in Siemens Biograph PET/MRI and PET/CT systems—a phantom study

**DOI:** 10.1186/s40658-019-0251-1

**Published:** 2019-08-05

**Authors:** Silje Kjærnes Øen, Lars Birger Aasheim, Live Eikenes, Anna Maria Karlberg

**Affiliations:** 1 Department of Circulation and Medical Imaging, Norwegian University of Science and Technology, Postbox 8905, N-7491 Trondheim, Norway; 2 Department of Radiology and Nuclear Medicine, St. Olavs University Hospital, Olav Kyrres gt 17, N-7006 Trondheim, Norway

**Keywords:** PET/CT, PET/MRI, CNR, Detectability

## Abstract

**Background:**

The technology of modern positron emission tomography (PET) systems continuously improving, and with it the possibility to detect smaller lesions. Since first introduced in 2010, the number of hybrid PET/magnetic resonance imaging (MRI) systems worldwide is constantly increasing. It is therefore important to assess and compare the image quality, in terms of detectability, between the PET/MRI and the well-established PET/computed tomography (CT) systems. For this purpose, a PET image quality phantom (Esser) with hot spheres, ranging from 4 to 20 mm in diameter, was prepared with fluorodeoxyglucose and sphere-to-background activity concentrations of 8:1 and 4:1, to mimic clinical conditions. The phantom was scanned on a PET/MRI and a PET/CT system for both concentrations to obtain contrast recovery coefficients (CRCs) and contrast-to-noise ratios (CNRs), for a range of reconstruction settings. The detectability of the spheres was scored by three human observers for both systems and concentrations and all reconstructions. Furthermore, the impact of acquisition time on CNR and observer detectability was investigated.

**Results:**

Reconstructions applying point-spread-function modeling (and time-of-flight for the PET/CT) yielded the highest CRC and CNR in general, and PET/CT demonstrated slightly higher values than PET/MRI for most sphere sizes. CNR was dependent on reconstruction settings and was maximized for 2 iterations, a pixel size of less than 2 mm and a 4 mm Gaussian filter. Acquisition times of 97 s (PET/MRI) and 150 s (PET/CT) resulted in similar total net true counts. For these acquisition times, the smallest detected spheres by the human observers in the 8:1 activity concentration was the 6-mm sphere with PET/MRI (CNR = 5.6) and the 5-mm sphere with PET/CT (CNR = 5.5). With an acquisition time of 180 s, the 5-mm sphere was also detected with PET/MRI (CNR = 5.8). The 8-mm sphere was the smallest detected sphere in the 4:1 activity concentration for both systems.

**Conclusion:**

In this experimental study, similar detectability was found for the PET/MRI and the PET/CT, although for an increased acquisition time for the PET/MRI.

## Background

In combination with structural imaging modalities such as computed tomography (CT) or magnetic resonance imaging (MRI), positron emission tomography (PET) is a versatile modality, which can acquire both qualitative and quantitative functional images. The modality plays an increasing role in detection, diagnosis, and staging of different types of cancer. With technological advances over the recent years, such as time-of-flight (TOF) capability, point spread function (PSF) modeling, better reconstruction algorithms, and improved detector design and materials, PET image quality and resolution have improved substantially, and with it the capability to detect and quantify smaller lesions [1]. The existing international standard for assessing PET image quality, National Electrical Manufacturers Association (NEMA) NU 2-2018 [2], does however not assess uptake volumes below 10 mm in diameter. Current PET systems are more than capable of detecting lesions smaller than this, and it is therefore a need for further evaluation of image quality, in terms of detectability, in current state-of-the-art systems by using uptake volumes below 10 mm in diameter [3–5]. This is especially important not only for the more recently introduced PET/MRI systems, but also for newly introduced digital PET/CT systems, where experimental setups assessing detection limits have not been previously performed. Qualitative evaluation of PET detectability is normally performed by human observers, using predefined scores to assess the detectability, while quantitative approaches most commonly measure the contrast-to-noise ratios (CNRs) [3, 4].

In clinical studies, efforts have been made comparing detection rates (in terms of number of lesions detected) between PET/MRI and PET/CT systems, and human observer studies have demonstrated equivalent detection rates in most types of cancers [6–8]. Exceptions include prostate cancer, bone metastases, and cerebrospinal lesions where PET/MRI might have advantages over PET/CT and small lung lesion detection where PET/CT with diagnostic CT has been reported superior to PET/MRI [6–8]. However, there is limited information regarding PET detection limits in terms of lesion size in the clinical studies. Furthermore, biological factors and attenuation correction issues will bias the results in clinical comparisons, making the interpretations of the results more complex than in experimental studies.

Only a few studies have investigated and optimized detectability in PET/CT systems, for sphere sizes smaller than 10 mm in an experimental setting. Hashimoto et al. [4] assessed the detectability using a NEMA body phantom with sphere diameters of 4 to 37 mm and a sphere-to-background ratio of 8:1 and detected hot spheres down to 6 mm with clinical scan times (2 min). They also found that the detectability index (similar to CNR in this study) and recovery coefficient were increased with TOF and for the smallest voxel size (2 mm), for spheres smaller than 10 mm in diameter. Other studies have also shown that reconstruction advances, such as TOF, PSF, and smaller voxel sizes can be used to improve the detectability [4, 9–21]. Using a Jaszczak phantom, Adler et al. [3] demonstrated that hot spheres down to 4–6 mm could be detected in a range of currently available PET/CT imaging systems employing clinical scan times (2–4 min). For the three clinically available PET/MRI systems, NEMA image quality results have recently been reported by Boellaard et al. [22] but does however not include detectability of small uptake volumes.

The purpose of this study was to experimentally investigate and compare the detectability of small uptake volumes (≥ 4 mm diameter) for the Biograph mMR PET/MRI system and the Biograph mCT PET/CT system, both manufactured by Siemens Healthcare (Erlangen, Germany). A PET image quality phantom (Esser PET phantom) was used for the calculation of contrast recovery coefficient (CRC) and CNR for hot spheres ranging from 4 to 20 mm diameters, for different sphere-to-background activity concentrations and for a range of different reconstruction settings. This quantitative approach was complemented with a blinded human observer study, where three observers scored the detectability of each sphere in all reconstructed PET images.

## Methods

### Phantom

**Fig. 1:**
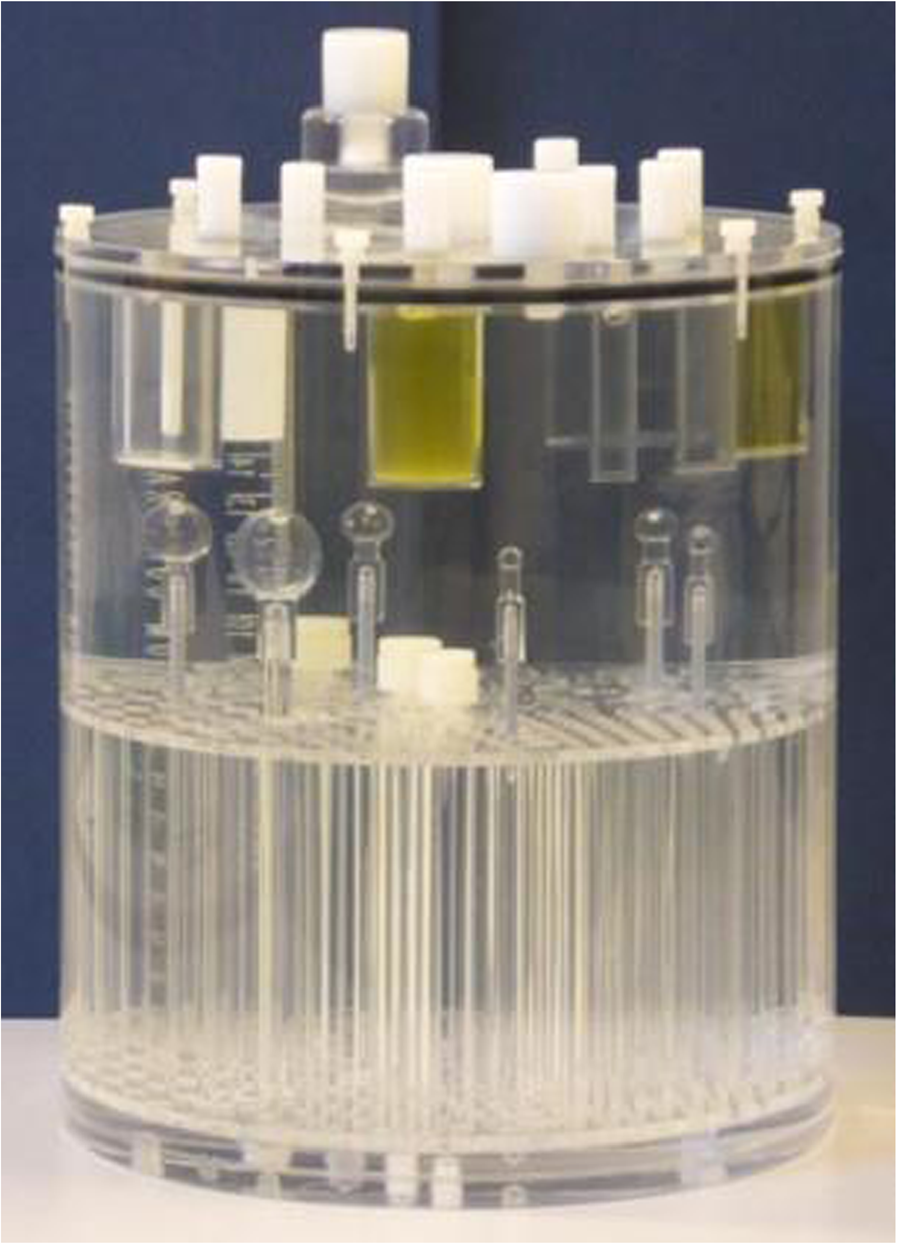
Esser PET phantom with additional coplanar hot spheres of 4, 5, 6, 8, 12, and 20 mm, attached to the bottom of the phantom

The phantom utilized in this study was an Esser PET phantom model PET/FL/P with lid model PET/LID/FL and additional hollow sphere models ECT/HS/SET6 and ECT/MI-HS/SET4 (Fig. 1) (Data Spectrum Corporation, Hillsborough, NC, USA). The phantom has a cylindrical shape with an inner diameter of 20 cm and an inner height of 19 cm, with a cold rod insert and a lid with seven protruding cold contrast cylinders (containing teflon, oil, air, and water) with outer diameters ranging from 8 to 25 mm. These inserts were only used for image registration purposes. Six coplanar hollow spheres with inner diameters of 4, 5, 6, 8, 12, and 20 mm were mounted in the phantom to represent the small uptake volumes of interest for this study.

### Data acquisition

The phantom was scanned with two different sphere-to-background activity concentrations (8:1 and 4:1) using fluorodeoxyglucose (^18^F-FDG) (Table 1). The activity concentrations were selected to mimic activity ranges typically found in clinical scans. For each concentration, acquisitions were first performed on the Biograph mMR (version VE11P) (Siemens Healthcare, Erlangen, Germany) followed by acquisitions on the Biograph mCT (version syngo MI.PET/CT 2012A) (Siemens Healthcare, Erlangen, Germany).

**Table 1:** Calculated activity concentrations in background (*C*_B,0_) and in hot spheres (*C*_H,0_), total activity (*A*_0_) at scan start, and time between scanning (Δ*t*) at the PET/MRI and the PET/CT

	Sphere to background			
	8:1		4:1	
	PET/MRI	PET/CT	PET/MRI	PET/CT
*C*_B,0_ [kBq/mL]	3.5	3.1	3.9	3.4
*C*_H,0_ [kBq/mL]	28.6	25.1	15.4	13.7
*A*_0_ [MBq]	20.3	17.8	21.9	19.4
Δ*t* [min]	20		19	

The PET/MRI protocol included a Dixon scan (TR 4.14 ms, TE1 1.28 ms, TE2 2.51 ms, field of view (FOV) 265 × 500 mm^2^, flip angle 10°, slice thickness 2.02 mm) and a 10-min (2 times the clinical acquisition time) listmode PET scan acquired in one bed position. The phantom was positioned in the center of the transaxial MRI FOV, in a low-attenuating foam phantom holder. The PET/CT protocol included a CT scan (reference tube current-exposure time product 200 mAs, peak tube voltage 120 kV, slice thickness 3 mm, collimation 64 × 0.6 mm, rotation time 1 s, pitch 0.9) along with a 5-min (2 times the clinical acquisition time) listmode PET scan acquired in one bed position. The phantom was positioned in the same holder as in the PET/MRI, in the center of the transaxial FOV. The PET/CT scans started 20 min (8:1 activity concentration) and 19 min (4:1 activity concentration) after the PET/MRI scans.

### PET detectors

Both the mMR and the mCT detectors consist of lutetium oxyorthosilicate (LSO) crystals of 4 × 4 × 20 mm. The mMR has an axial PET FOV of 25.8 cm, a transaxial FOV of 58.8 cm, and a detector ring diameter of 65.6 cm [23]. The integrated whole-body MR is a 3 Tesla niobium-titanium magnet. The mCT has an axial PET FOV of 22.1 cm and a transaxial FOV of 70 cm, the detector ring diameter is 84.2 cm and a 64-slice CT is integrated [24].

### Phantom attenuation correction

For clinical protocols, attenuation correction maps (AC maps) are generated automatically; CT-based AC (CTAC) is based on the bilinear conversion of CT values to attenuation values for the specific photon energy 511 keV, while MR-based AC (MRAC) normally is based on the aforementioned Dixon sequence. The Dixon sequence enables separation of water and fat signal from a human body and allows segmentation of fat, soft-tissue, and air as well as lung tissue. The Dixon-based AC map is generally not suited for AC of phantoms due to the risk of artifacts in MR images at high magnetic field strengths when scanning large water-filled phantoms [22, 25]. For this reason, the CTAC map was also used for AC of PET/MRI data. This means that uncertainties due to attenuation correction, common in clinical PET/MRI imaging, can be disregarded. To enable CTAC at the PET/MRI, a CTAC map of the phantom was first multiplied with 10,000 to get the same scale as the MRAC maps from the PET/MRI system. Then, the corresponding CTAC image was rigidly registered to the in-phase image from the Dixon sequence, and the CTAC map was transformed with the resulting transformation matrix to match the in-phase image and hence the MRAC map. The multiplication and registrations were performed with the software Aliza 1.38.2 (Aliza Medical Imaging, Bonn, Germany). To enable import of the CTAC maps to the PET/MRI system, the pixel data of the MRAC maps was replaced by the pixel data of the CTAC maps.

### PET reconstructions

The PET data was reconstructed with a range of different reconstruction settings (Table 2). 3D iterative reconstruction algorithm was employed (ordered subset expectation maximization (OSEM)), with and without PSF modeling (both systems) as well as with and without TOF (PET/CT). The number of iterations varied from 1 to 8, and the pixel size varied from ~ 1 mm to ~ 6 mm. Furthermore, Gaussian filter of 4 mm and 2 mm was applied, in addition to reconstructions without filter (all-pass). The same number of net true counts was used for a direct comparison between the PET/CT and PET/MRI. PET/CT listmode data was reconstructed for the first 150 s, as this is the typical clinical acquisition time/bed on this system, based on guidelines for PET/CT ^18^F-FDG examinations [26]. The same net true counts were reached at 97 s with the PET/MRI (due to the higher sensitivity of the PET/MRI system), and this time was therefore used for the direct comparisons between the systems. In order to compare CNR and detectability improvements over time, reconstructions with increasing time frames of 1 min were also performed, from 1 to 5 min for the PET/CT and from 1 to 10 min for PET/MRI. This corresponds to twice the typical acquisition times/bed for each system. On-scanner software for each system was used for the reconstructions, which included CT-based attenuation corrections for all reconstructions, as well as corrections for decay, detector normalization, randoms, and scatter.

**Table 2:** Evaluated reconstruction settings for the phantom PET scans for both 8:1 and 4:1 activity concentration

System	Reconstruction algorithm	Iterations	Subsets	Matrix [pixels]	Voxel size [mm]	PET acquisition time [s]	Filter
PET/CT	OSEM	1–8	24	400 × 400	2.0 × 2.0 × 2.0	150	4 mm Gauss
	OSEM&PSF						
	OSEM&TOF		21				
	OSEM&TOF&PSF						
		3		128 × 128	6.4 × 6.4 × 2.0		
				200 × 200	4.1 × 4.1 × 2.0		
				256 × 256	3.2 × 3.2 × 2.0		
				512 × 512	1.6 × 1.6 × 2.0		
		1–8		400 × 400	2.0 × 2.0 × 2.0		2 mm Gauss
							No filter
		3				60, 120, 180, 240, 300	4 mm Gauss
PET/MRI	OSEM	1–8	21	344 × 344	2.1 × 2.1 × 2.0	97	4 mm Gauss
	OSEM&PSF						
		3		128 × 128	5.6 × 5.6 × 2.0		
				172 × 172	4.2 × 4.2 × 2.0		
				256 × 256	2.8 × 2.8 × 2.0		
				512 × 512	1.4 × 1.4 × 2.0		
				344 × 344	2.1 × 2.1 × 2.0		2 mm Gauss
							No filter
						60, 120, 180, 240, 300, 360, 420, 480, 540, 600	4 mm Gauss

### Image analysis

#### Prompts, randoms, trues, and scatter fraction

The number of prompts, randoms, and total net trues was extracted from the sinogram headers at each system for the acquisition times that yielded the same number of total net trues, for both activity concentrations. The estimated scatter fraction was extracted from DICOM headers.

#### CRC and CNR

**Fig. 2:**
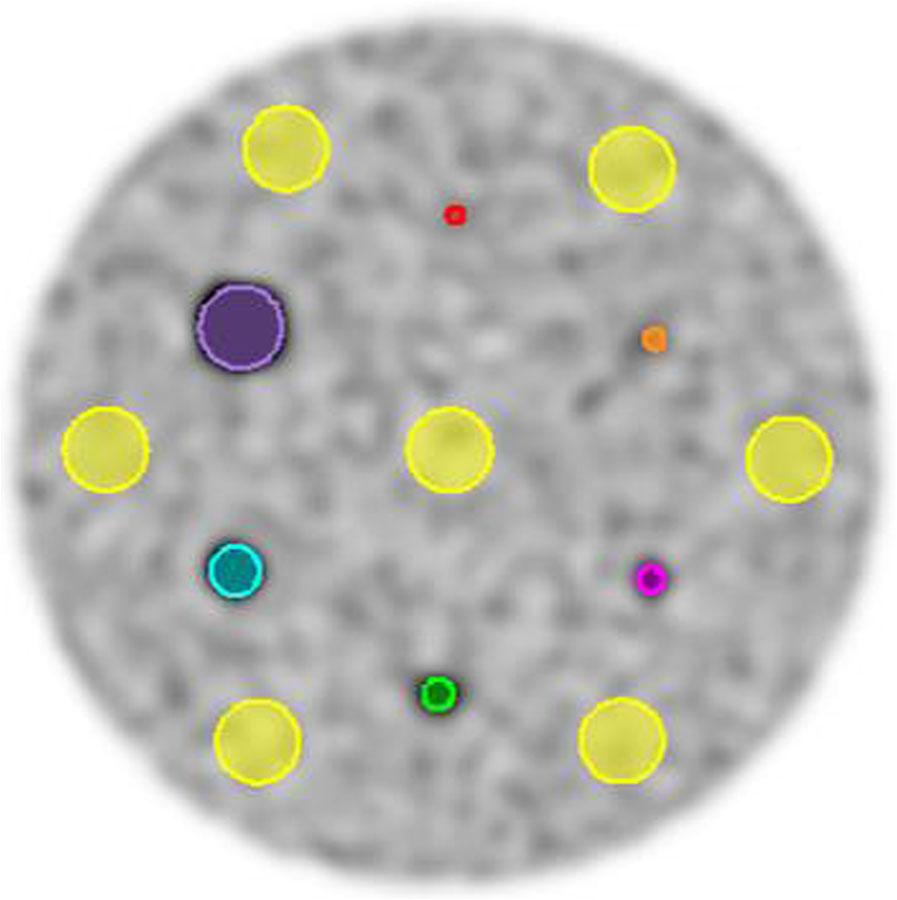
Spherical VOIs placed over the hot spheres in OSEM with PSF and TOF with activity concentration of 8:1 (3 iterations, 2 mm voxel size) (purple: *d* = 20 mm, cyan: *d* = 12 mm, green: *d* = 8 mm, pink: *d* = 6 mm, orange: *d* = 5 mm, red: *d* = 4 mm) and seven spherical background VOIs (yellow: *d* = 20 mm)

To perform quantitative analyses, spherical volumes of interest (VOIs) were placed over the hot spheres of the phantom, with the same size as the inner volume of the spheres. In addition, seven spherical background VOIs (*d* = 20 mm) were centered in the same transaxial plane as the hot spheres, one in the central part and six uniformly distributed close to the edge of the phantom (Fig. 2). The VOIs were placed on a CT image and the set of VOIs were manually adjusted to fit the activity in the PET images for each of the four scan sessions (two activity concentrations on two systems) and subsequently copied to all the remaining reconstructions of the same scan session.

**Fig. 3:**
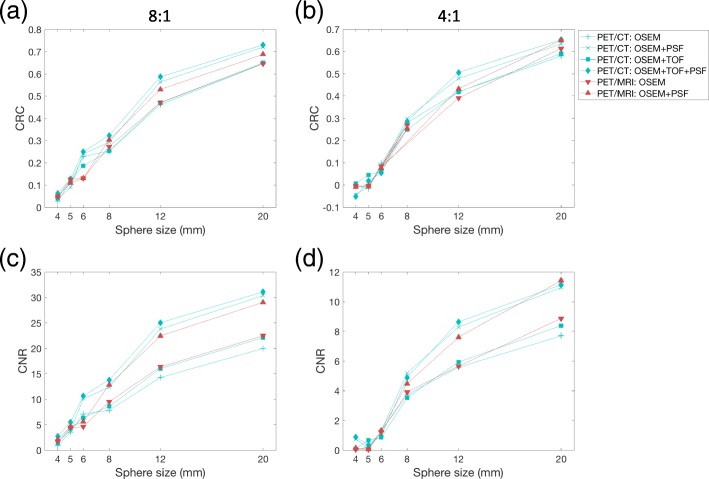
Contrast recovery coefficients (CRCs) and contrast-to-noise ratios (CNRs) for the PET/CT and PET/MRI systems. CRC vs sphere size for **a** 8:1 and **b** 4:1 activity concentrations, and CNR vs sphere size for the **c** 8:1 and **d** 4:1 activity concentrations (all reconstructions with 3 iterations and 2 mm voxel size)

CRC and CNR were calculated to quantitatively compare the detectability between the systems, for different reconstruction settings. CRC provides information of how accurately the system reproduces the true activity concentration in a specific volume, but gives little or no information about the visibility of a specific uptake volume, like CNR. CRC was calculated as defined by Kessler et al. [27] and NEMA [2]:

1 $$ \mathrm{CRC}=\frac{\left(\raisebox{1ex}{${C}_H$}\!\left/ \!\raisebox{-1ex}{${C}_B$}\right.\right)-1}{\left(\raisebox{1ex}{${a}_H$}\!\left/ \!\raisebox{-1ex}{${a}_B$}\right.\right)-1} $$

where *C*^*H*^ is the average counts in the hot sphere VOI, and *C*^*B*^ is the average counts of the background VOIs, while *a*^*H*^ and *a*^*B*^ is the activity concentration in the hot sphere and the background, respectively. While CNR was calculated according to [28]:

2 $$ \mathrm{CNR}=\frac{\left|{C}_H-{C}_B\right|}{SD_B} $$

where *SD*_*B*_ is the standard deviation of the counts in the background VOIs.

For the direct comparisons of CRC and CNR for different sphere sizes, the 344 × 344 matrix size was used for PET/MR images and the 400 × 400 matrix size for PET/CT images, as this corresponds to approximately the same pixel size. The relationship between CNR and number of iterations, pixel size, and filter was further evaluated for the smallest sphere with CNR > 5 (Rose criterion) on both systems for the acquisitions with similar number of true counts in the 8:1 concentration**.**

#### Detectability—human observer study

To qualitatively evaluate the detectability of the hot spheres, three observers (physicists experienced in the field of medical imaging) individually scored the detectability of each hot sphere in all PET reconstructions from both systems. The PET images were presented to the observers in random order, blinded for the reconstruction settings. The detectability of the spheres was scored from 0 to 2, similar to the score system used by Adler et al. [3], where 0 was not detectable, 1 was visible, but comparable to noise, and 2 was clearly visible. The images were evaluated in *syngo*.via (software version VB10/30) (Siemens Healthcare, Erlangen, Germany). A sphere was defined as detected if the sum of the scores from the three observers were 3 or higher, with the restriction that no observer gave a score of 0, and all remaining spheres were defined as non-detected.

#### CNR and detectability

CNR and detectability improvements with increased acquisition times were evaluated for spheres with CNR < 5 (Rose criterion) on any system in the 8:1 concentration (for the 150 s PET/CT and 97 s PET/MRI reconstructions), in order to evaluate whether spheres with CNR < 5 would improve CNR and become detectable with increasing scan time. Furthermore, the relation between CNR and detectability in the human observer study was also investigated and compared to the Rose criterion, which states that an object is detectable if CNR is above 3–5, depending on characteristics such as object size and shape, edge sharpness, viewing distance, and observer experience [29].

#### Statistics

To evaluate inter-reader agreement, Kappa statistics were performed (with Stata/MP 15.1, StataCorp LLC, USA). Cohen/Conger’s Kappa was used in this study, calculated by the kappaetc package by Daniel Klein, based on formulas in [30]. Weighted kappa was used and the weight was 1 for perfect agreement, 0.5 for a difference in score of one, and 0 for a difference in score of two.

## Results

### Prompts, randoms, trues, and scatter fraction

The total number of prompts, randoms, and trues and estimated scatter fraction, for acquisition times yielding the same number of total net trues, are demonstrated in Table 3, for both activity concentrations. The number of prompts and randoms, as well as scatter fraction, were higher for the PET/MRI compared to the PET/CT.

**Table 3:** The number of prompts, randoms, total net trues, and estimated scatter fraction, for the phantom scans (PET/CT 150 s acquisition, PET/MRI 97 s acquisition), for activity concentrations of 8:1 and 4:1

	Sphere to background			
	8:1		4:1	
	PET/CT	PET/MRI	PET/CT	PET/MRI
Prompts (MCts)	20.9	22.2	22.6	24.2
Randoms (MCts)	1.8	3.1	2.0	3.5
Total net trues (MCts)	19.1	19.2	20.7	20.7
Scatter fraction (%)	30.1	31.1	29.8	30.9

### CRC and CNR

For most of the largest spheres, PET/MRI and PET/CT yielded similar CRC and CNR for the OSEM-only reconstructions, while a slight increase for PET/CT was seen with the OSEM+PSF reconstructions (Fig. 3). The addition of TOF at the PET/CT did also cause a small increase in CRC and CNR. In the 8:1 concentration, CNR > 5 (Rose criterion) was reached for the 6-mm sphere for both systems, and this sphere was therefore used in the further evaluations of CNR.

**Fig. 4:**
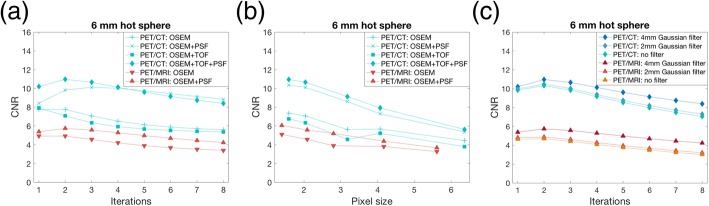
Contrast-to-noise ratios (CNRs) for the PET/CT and PET/MRI systems, for **a** increasing number of iterations (pixel size ~ 2 mm) and **b** increasing transaxial pixel sizes (3 iterations), and **c** CNR vs iterations for different filter settings (PET/CT: OSEM+TOF + PSF, PET/MRI: OSEM+PSF). All results are for the 6-mm hot sphere in the 8:1 activity concentration

The effect of number of iterations, pixel size, and filter on CNR is shown in Fig. 4. Maximum CNR was reached with two iterations for both imaging systems, with PSF for PET/MRI and for the combination of PSF and TOF for PET/CT (Fig. 4a). For PET/CT, it was seen that reconstructions with PSF only needed more iterations to converge compared to reconstructions with both PSF and TOF. Small pixel sizes (< 2 mm) yielded the highest CNR for both systems (Fig. 4b). A Gaussian filter of 4 mm FWHM resulted in the highest CNR for both systems (Fig. 4c).

### Detectability—human observer study

With similar number of true counts for the two systems, the 6-mm sphere was the smallest detected sphere with PET/MRI in the 8:1 activity concentration. The 6-mm sphere was detected in all reconstructions, except for four iterations or more, or a pixel size of 3 mm, in the reconstructions without PSF. The 5-mm sphere was detected with PET/CT in the 8:1 activity concentration (for some reconstructions including TOF, with more than two iterations, and a pixel size below 4 mm), while in the 4:1 activity concentration, the 8 mm sphere was the smallest detected sphere for both systems and in all reconstructions with a few exceptions (PET/MRI: OSEM, 1 iter, 2 mm pixel size; OSEM+PSF, 3 iter, pixel size > 4 mm; PET/CT: OSEM+TOF, 3 iter, 6 mm pixel size). Modifying the filter setting did not yield detection of smaller spheres.

The percentage agreement between the observers for all reconstructions were 0.95% (95% C.I. 0.94–0.96) and Kappa was 0.89 (95% C.I. 0.88–0.91), which correspond to almost perfect agreement according to definitions by Landis et al. [31].

### Detectability and CNR

**Fig. 5:**
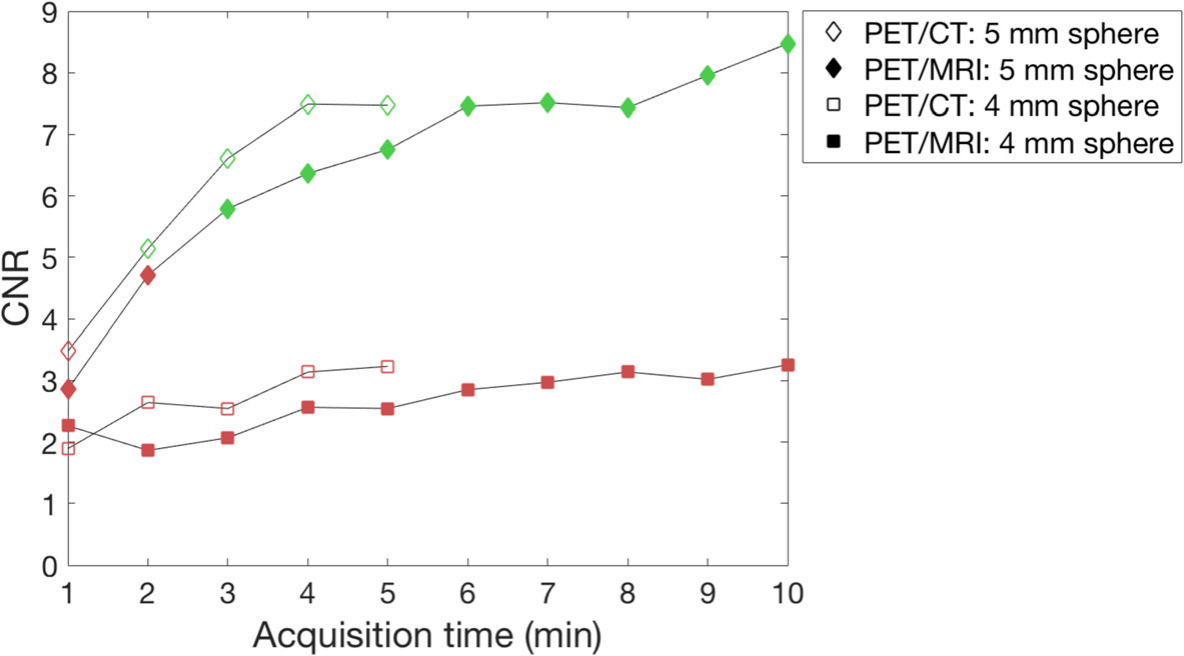
Contrast-to-noise ratio (CNR) for increasing acquisition times on the PET/MRI (1–10 min, OSEM with PSF, 3 iterations, and 2 mm voxel size) and the PET/CT (1–5 min, OSEM with PSF and TOF, 3 iterations, 2 mm voxel size) for the 4-mm and 5-mm spheres in the 8:1 activity concentration. Spheres detected in the human observer study are presented in green, while non-detected spheres are presented in red

**Fig. 6:**
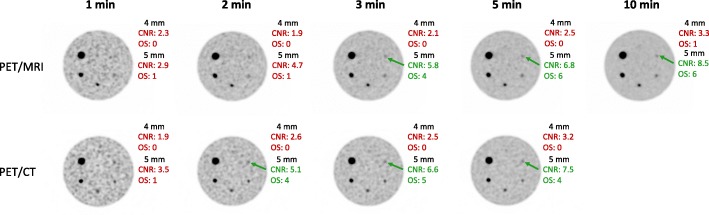
PET images from the PET/MRI and PET/CT with 8:1 activity concentration for increasing acquisition times. Contrast-to-noise ratio (CNR) and observer scores (OS) for the hot spheres with a diameter of 4 mm and 5 mm are presented in red when the sphere was defined as non-detected and in green when the sphere was defined as detected. PET/MRI images are reconstructed with OSEM with PSF, while PET/CT images are reconstructed with OSEM with PSF and TOF, all with 3 iterations and 2 mm voxel size

The effect of increased acquisition times on CNR and detectability were evaluated for the 4 and 5 mm spheres, as these spheres had a CNR below 5 on the PET/MRI from the acquisitions with similar number of true counts. The results demonstrated that the 5-mm sphere was the smallest detected sphere for both systems, requiring a 3-min acquisition on the PET/MRI and a 2-min acquisition on the PET/CT (Fig. 5), which is less than the clinical acquisition times used at our hospital. Corresponding PET images for increasing acquisition times are presented in Fig. 6.

**Fig. 7:**
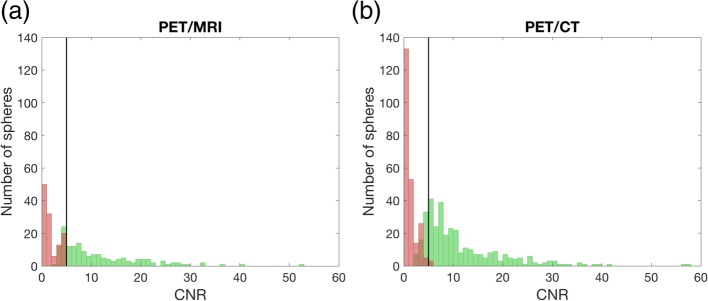
Histograms of contrast-to-noise ratio (CNR) for detected (green) and non-detected (red) spheres for **a** PET/MRI and **b** PET/CT for all the reconstructions with similar number of true counts for the two systems. The black line represents CNR = 5 (Rose criterion). There are twice as many reconstructions for the PET/CT than for the PET/MRI due to the TOF option

The distribution of CNR for detected and non-detected spheres is illustrated in Fig. 7, and the distribution was similar for PET/MRI and PET/CT. CNR was above 3 for all detected spheres, except for the 8-mm sphere in one PET/MRI reconstruction and seven PET/CT reconstructions which yielded CNR between 2.3–3.0. CNR was below 5 for the non-detected spheres, except for the 5-mm sphere which had a CNR of 5.5–5.6 in three PET/CT reconstructions.

## Discussion

In this study, the detectability of Siemens PET/MRI and PET/CT systems were evaluated and compared in an experimental setting, both quantitatively and by human observers. Spheres down to 5 mm were considered detected by both systems, for shorter acquisition times than normally used in clinical routine.

In general, the PET/CT showed slightly increased CRC and CNR for the OSEM+PSF(+TOF) reconstructions compared to PET/MRI. CRC and CNR increased when PSF was incorporated in the reconstruction, and a further increase was found when both PSF and TOF were included. Improvements by PSF and TOF have also been demonstrated previously [4, 13, 15–17, 19, 20], and this indicates that these algorithms should be used to increase the possibility of detecting small lesions. PSF is a geometry correction that provides higher and more uniform spatial resolution over the transaxial FOV and will therefore have the most impact at the outer edges of the FOV [32]. The inclusion of TOF increases the signal-to-noise ratio (SNR) in the image, and the increase is proportional to the size of the imaging object [32]. Thus, PSF and TOF will probably have a larger effect in clinical patient scans than for the phantom in this study, since the Esser phantom has a smaller diameter than a typical patient. The TOF option was only available on the PET/CT system, since the timing resolution of the avalanche photodiode (APD) detectors in the Siemens Biograph PET/MR system is too slow for TOF. It should be noted, however, that PSF may cause edge artifacts (Gibbs artifacts) and should be used with caution in quantitative analysis of sub-centimeter lesions [10, 33].

The number of iterations and post-filtration had a small effect on CNR. The maximum CNR for two iterations corresponds to previous results on image quality for the same systems [16]. More iterations improve the quantitative accuracy of the image but does also increase the background noise [34]. However, new algorithms, such as Bayesian penalized likelihood algorithms, maintain high image quality with more iterations by suppressing noise with a penalty function. The penalty function takes prior knowledge into account and warrants convergence without amplifying noise, resulting in increased activity measures and improved detection of small objects [32, 34–40]. Out of the evaluated filter settings, a Gaussian filter of 4 mm FWHM should be preferred as CNR then was maximized. The choice of pixel size had a larger impact on CNR, especially for the PET/CT. Current guidelines for whole-body ^18^F-FDG PET recommend pixel sizes of 3.0–4.0 mm [26]. With new technology and recent developments, modern PET systems allow pixel sizes down to 1 mm, but these are rarely used. Our study shows increased CNR and detectability of small uptake volumes for a decreased pixel size, in accordance with other studies [3, 11, 12, 21, 41]. This, in addition to the use of a 10-mm sphere as the smallest sphere in the NEMA standard for assessing PET quality, implies that there is a need for upgrading current PET imaging guidelines and standardized imaging quality phantoms to meet the performance of modern PET systems.

The human observer study revealed lower detection limits in PET/CT images (5 mm) compared to PET/MRI images (6 mm) for the 8:1 activity concentration for acquisitions with similar number of true counts, while the same detection limit of 8 mm was found for both systems for the 4:1 activity concentration. For an increased acquisition time on the PET/MRI, the detectability was however comparable between the systems, indicating that longer scan times are required on the Siemens PET/MRI to obtain the same detectability as the Siemens PET/CT. In clinical PET/MR imaging, the acquisition times should probably be exceeded even further, since more factors will influence image quality and detectability, such as the slightly increased scatter fraction of PET/MRI that will be further increased for a patient that is larger than the phantom, increased attenuation as the patient’s arms are positioned alongside the body, and smaller bed overlap in multi-bed scans on PET/MRI compared to PET/CT.

As TOF slightly increased CRC and CNR, it was also required to detect the 5 mm sphere in the human observer study. Despite quantitative improvements also with the use of PSF, the detectability was in general not improved with PSF in the observer study. The use of a relatively large filter, such as the 4 mm Gaussian filter, could explain why the 4-mm sphere was not detected. However, the sphere was not detected with a 2-mm Gaussian filter or when the filter was omitted.

There are several factors that limit the spatial resolution in PET, but one of the main limitations is the size of the detector element (crystal) [42]. Other factors include positron range and acollinearity factors (which cannot be reduced), decoding errors, crystal penetration, and reconstruction algorithm. Both the PET/MRI and PET/CT systems have lutetium oxyorthosilicate (LSO) crystals with a dimension of 4 × 4 × 20 mm. The theoretical spatial resolution can be estimated for a given system [42], and with the effective positron range for ^18^F in water [43], this yields 4.5 and 4.7 mm full width at half maximum (FWHM) in isocenter for the PET/MRI and PET/CT, respectively. The detection limits found in this study therefore seem to approach the actual spatial resolution limits of the systems, also for clinical scan times.

The correlation between the detectability in the human observer study and CNR showed a similar distribution for PET/MRI and PET/CT, and most of the detected spheres had a CNR above 3 and most of the non-detected spheres had a CNR below 5, which is in concordance with the Rose criterion. These results indicate that the scoring system utilized in this study seems functional for this type of experimental setting. Adler et al. [3] used a more conservative limit for detected spheres (score = 5), which also agreed with the Rose criterion. However, they defined some spheres as “neither observed nor not observed” and excluded these spheres from the analysis, which might have influenced the result. The Rose criterion will change with the sphere size and matrix size, but was not adjusted for in this study.

Several technical factors influence the quantitative measurements in clinical PET/MRI data, but could be neglected in this study by using a phantom with CT-based AC for both systems. For clinical scans at the Siemens PET/MRI system, AC is based on segmentation of tissue, providing predefined attenuation coefficients for soft tissue, fat, lung tissue, and air, and bone is included by co-registration with a bone atlas. This can cause quantification and registrations errors, influencing the PET images [44, 45]. In addition, the flexible body surface coils are not accounted for in clinical AC PET images and may lead to a regionally dependent bias [46]. Furthermore, respiratory motion can lead to PET image blurring, artifacts, and tracer uptake quantification errors in general [47], but this affects both PET/MRI and PET/CT data, and motion correction methods are improving [47–50]. A review study by Spick et al. [6] summarized 46 studies (including 2340 patients) and found that the PET/MRI and PET/CT provide comparable diagnostic information for most types of cancer despite both technical and operational issues.

The results of this study are not directly transferable to clinical practice due to the use of a phantom with spheres in fixed positions; hence, the observers know where to look for the spheres. Furthermore, the observers were physicists and not physicians, but the kappa statistics showed almost perfect agreement between the readers. A total score of 3 was considered detected in this study, since we wanted to investigate detectability, and not if the spheres were clearly visible.

Another limitation of this study is the size of the Esser phantom, which is more representative for brain imaging, than for whole body imaging. More noise would have been present with a larger phantom, which for instance could have increased the benefit of PSF and TOF. Furthermore, the limited number of sphere sizes utilized in this study could have an impact on the results, as lower detection limits could have been obtained for the 4:1 activity concentration with a 7-mm sphere size. Another limitation is that only one scan per activity concentration was analyzed for each system. To reduce the possible statistical errors this could have caused, the mean values of the activity in the hot spheres were used in the analyses.

## Conclusion

Similar detectability performance was found for PET/CT and PET/MRI, although for an increased acquisition time on the PET/MRI. Hot spheres as small as 5 mm were detected with a 2-min PET/CT acquisition and a 3-min PET/MRI acquisition, corresponding to a CNR of 5.1 and 5.8, respectively. Reconstruction improvements, such as TOF and/or PSF, should be used to increase the possibility of detecting small lesions. Furthermore, smaller pixel sizes than recommended by current guidelines should be considered for oncological ^18^F-FDG scans.

## Data Availability

The datasets used and analyzed during the current study are available from the corresponding author on reasonable request.

## Abbreviations

^18^F-FDG: Fluorodeoxyglucose
AC: Attenuation correction
APD: Avalanche photodiode
CNR: Contrast-to-noise ratio
CRC: Contrast recovery coefficient
CT: Computed tomography
FOV: Field of view
FWHM: Full width at half maximum
LSO: Lutetium oxyorthosilicate
MRI: Magnetic resonance imaging
NEMA: National Electrical Manufacturers Association
OSEM: Ordered subset expectation maximization
PET: Positron emission tomography
PSF: Point spread function
SNR: Signal-to-noise ratio
TOF: Time-of-flight
VOI: Volume of interest
